# Integrated bioinformatic analysis identifies UBE2Q1 as a potential prognostic marker for high grade serous ovarian cancer

**DOI:** 10.1186/s12885-021-07928-z

**Published:** 2021-03-04

**Authors:** Rachel Topno, Ibha Singh, Manoj Kumar, Pallavi Agarwal

**Affiliations:** 1 Amity Food and Agriculture Foundation, Amity University Uttar Pradesh, Sector 125, Noida, 201313 India; 2 Present Address: Institut de Génétique Humaine, Montpellier, France; 3 Amity Institute of Molecular Medicine and Stem Cell Research, Amity University Uttar Pradesh, Sector 125, Noida, 201313 India

**Keywords:** High grade serous ovarian cancer, UBE2Q1, WGCNA, B4GALT3, Breast Cancer

## Abstract

**Background:**

High grade serous ovarian cancer (HGSOC) accounts for nearly 60% of total cases of epithelial ovarian cancer. It is the most aggressive subtype, which shows poor prognosis and low patient survival. For better management of HGSOC patients, new prognostic biomarkers are required to facilitate improved treatment strategies and ensure suitable healthcare decisions.

**Methods:**

We performed genome wide expression analysis of HGSOC patient samples to identify differentially expressed genes (DEGs) using R based Limma package, Clust and other statistical tools. The identified DEGs were subjected to weighted gene co-expression network analysis (WGCNA) to identify co-expression patterns of relevant genes. Module trait and gene ontology analyses were performed to establish important gene co-expression networks and their biological functions. Overlapping the most relevant DEG cluster 4 with prominent WGCNA cyan module identified strongest correlation of UBE2Q1 with ovarian cancer and its prognostic significance on survival probability of ovarian cancer patients was investigated. The predictive value of UBE2Q1 as a potential biomarker was analysed by correlating its expression with 12-months relapse free survival of patients in response to platin/taxane, the standard first-line chemotherapy for ovarian cancer, and analysing area under the ROC curve.

**Results:**

An integrated gene expression analysis and WGCNA, identified UBE2Q1 as a potential prognostic marker associated with poor relapse-free survival and response outcome to platin/taxane treatment of patients with high grade serous ovarian cancer.

**Conclusions:**

Our study identifies a potential UBE2Q1 – B4GALT3 functional axis in ovarian cancer, where only the E2 conjugating enzyme showed a poor prognostic impact on the disease.

**Supplementary Information:**

The online version contains supplementary material available at 10.1186/s12885-021-07928-z.

## Background

Ovarian cancer (OC) is one of the most lethal gynaecological malignancies with a global age-standardized incidence rate of 6.6 and age-standardized mortality rate of 3.9 per 100,000 which is around 60% of incidence rate [1]. Major part of ovarian cancer mortality is attributed to the most aggressive high-grade serous subtype. The standard first line treatment regimen of ovarian cancer comprises of cytoreduction surgery and chemotherapy [2]. At the start of chemotherapy, most of the patients are responsive to paclitaxel and carboplatin combination therapy, however, over the time, they develop resistance against the drugs, which results in disease recurrence and a very low 5-year survival rate of 46% [3]. To identify the outcome of a primary treatment and/or future risk of disease recurrence in a patient, various biomolecules such as genes, proteins, lipids, glycolipids, glycoproteins, RNA, miRNA, LncRNA and other molecules are investigated as prognostic markers in the tumor tissue and the tumor micro-environment. These markers are important to stratify patients according to their risk groups and plan their treatment strategies and counselling aid [4, 5]. Further, such prognostic markers may contribute to the pathogenesis and progression of the disease thus opening up avenues for newer and better treatment strategies.

One of the main challenges with ovarian cancer is that it is diagnosed at later stages due to atypical symptoms and absence of effective diagnostic/screening methods. Further, robust prognostic markers to monitor the outcome of patient treatment are also lacking. Currently, CA125 and HE4 are suggested as prognostic biomarkers for ovarian cancer, however they lack specificity and sensitivity in monitoring disease progress [6].

In few reports, researchers have tried to identify probable prognostic biomarkers for ovarian cancer. In a study by Yang L et al., by studying differentially expressed genes (DEGs) related to different stages of ovarian cancer patients, the authors generated stage specific co-expression network modules and identified COL3A1, COL1A1, COL1A2, KRAS, and NRAS as candidate prognostic genes for ovarian cancer [7]. Using similar strategy, 4 M2 macrophage surface marker genes were found to be associated with poor prognosis of ovarian cancer patients [8] Researchers have also used WGCNA analysis to investigate molecular complexities of mucinous epithelial ovarian cancer and observe alterations in metabolism and steroid hormone biosynthesis processes [9]. Zhao et al. identified a lncRNA-based signature for survival prediction in ovarian cancer patients [10]. WGCNA analysis identified 33 lncRNAs in four modules which had relevant biological function and related with ovarian cancer clinical factors. Further, an optimal prognosis combination containing 5 lncRNAs (GAS5, HCP5, PART1, SNHG11, and SNHG5) was identified using a Cox-Proportional Hazard model [10]. Another study identified five lncRNA (LINC00664, LINC00667, LINC01139, LINC01419, and LOC286437) as independent risk factors for recurrence of ovarian cancer [11]. With respect to platinum chemoresistance in ovarian cancer, another study correlated expression of ADH1B, CDH11, VGLL3, and KIT with the drug resistance and suggested these genes as potential prognostic markers [12]. Eventhough these studies have identified a few candidates as prospective prognostic markers, a plethora of newly discovered genomic data from ovarian cancer patient samples are available, which could further facilitate identification of new prognostic biomarkers specific to ovarian cancer and can help in better therapy, management and stratification of the disease.

High throughput next generation sequencing data facilitates to develop gene co-expression networks, which simultaneously provides information about gene expression correlation and potential functional relationship. Study of such networks assists in understanding the biological system and exploring the relationship between the relevant functional genes. In this regard, weighted gene co-expression network analysis (WGCNA) is an in silico analytical tool which creates co-expression modules from available gene expression data, explores the signaling networks and may provide insights into the phenotypical indicators of tumor progression, grades and its metastasis. This method is already utilized to identify hub genes associated with clinical traits in a number of cancer types, such as pancreatic carcinoma and clear cell renal cell carcinoma [11]. Although certain studies are also reported in ovarian cancer, availability of large-scale expression data analysing tumor and normal samples demands a continuous need to evaluate this data and identify new molecules that may serve as potential prognostic markers.

In this study, we performed a genome wide expression analysis of publicly available GEO dataset GSE66957 (available online: https://www.ncbi.nlm.nih.gov/geo) comprising of 57 ovarian cancer patient tumor samples and compared it with 12 normal ovarian surface epithelial tissue samples. Using a fold-change difference threshold of > 2 fold, we identified 2607 differentially expressed genes in ovarian cancer in comparison to normal epithelial tissues. Using weighted gene co-expression network analysis, we established correlation patterns among differentially expressed genes in ovarian cancer. Module trait relationship between cancer and normal tissues was established and gene enrichment analysis was performed for major modules. Membrane signalling pathways, extracellular matrix structural components, immune signalling and protein degradation pathways predominated in the GO analysis of the relevant genes of major modules. An integrated gene expression analysis, both using DEGs and WGCNA, identified UBE2Q1, a yet less explored E2 ubiquitin-conjugating enzyme, as a potential prognostic marker associated with poor relapse-free survival and response outcome to platin/taxane treatment of patients with high grade serous ovarian cancer. The role of UBE2Q1 in embryo implantation and hormonal homeostasis in females makes this discovery intriguing and further studies will unravel how UBE2Q1 connects hormonal homeostasis with ovarian cancer [13].

## Methods

### Microarray data analysis

Gene expression profiles of GEO accession GSE66957 of ovarian cancer and normal ovarian tissue were obtained. This series includes 57 ovarian samples and 12 normal samples, based on GPL15048 Platform (Affymetrix Human GeneRosetta/Merck Human RSTA Custom Affymetrix 2.0 microarray). The raw data were normalized using the Robust Multiarray Average (RMA) method. A linear model was fit to the data using the limma package in R and the Empirical Bayes method was used to smoothen the standard errors. The method of Benjamini and Hochberg correction was applied to the *p* values. Genes were considered as differentially expressed if the adjusted p value is less than 0.01 (False discovery rate set to 1%) and |logFC| ≥ 2. Gene Probe ids for which annotations were not available were removed from the analysis. A heatmap was plotted with all the differentially expressed genes (DEGs). The DEGs were also clustered using program clust (http://clust.baselabujamous.com/Home.aspx), which filtered 427 genes in 5 clusters.

### Gene co-expression network construction

Gene co-expression network analysis was performed using the R package, WGCNA. The process is summarized as follows. The results from differential expression analysis were sorted and the expression for different probe ids representing the same gene were averaged for each gene. A matrix of pairwise correlations between all pairs of genes across all selected samples was constructed. Second, the soft-thresholding power was chosen as 9 using the pickSoftThreshold function of the WGCNA package to which co-expression similarity is raised to achieve consistent scale free topology in our dataset. Third, with the chosen power value, we performed step-by-step network construction and module detection with the following major parameters using the TOM matrix and the cutreeDynamic function: deepSplit of 2, cutHeight of 0.998 and mergeCutHeight of 0.25 while merging of similar modules. This procedure comprised calculation of network adjacencies and topological overlap dissimilarities, followed by scaling of topological overlap matrices and calculation of consensus topological overlap. Then, we built a hierarchical clustering dendrogram of gene expression data and performed adaptive branch cutting to identify modules. Some modules with similar expression profiles were merged, according to pre-defined parameters.

### Calculation of module-trait correlations and module preservation

The correlations among gene expression modules and clinical traits for our dataset was analysed choosing cancer status as the clinical trait. Modules having significant relationships with the relevant traits were labelled using a conventional colour scheme. An intramodular analysis of gene trait significance and module membership of the genes in the 3 significant modules with a correlation > 0.9 was performed.

### Network visualization

The network, with edges having weight > 0.7 was then exported to Cytoscape for visualization. Unconnected node pairs with single edges and isolated nodes were removed for a simplified view of the full network. Network parameters were calculated using the Cytoscape plugin, NetworkAnalyzer. The node colours correspond to the modules obtained using WGCNA.

### GO analysis

In order to perform gene ontology, the functional annotation chart for all the filtered genes containing the GO terms and their enrichment were obtained from DAVID database. The R package GOPlot was used to combine the results from the gene annotation analysis and the gene expression analysis and visualize them in the form of a circular bar plot.

### Kaplan-Meier survival analysis

Progression free or relapse free survival of ovarian and breast cancer patients, respectively, was associated with gene expression using online database (http://kmplot.com/analysis/) [14, 15] and Kaplan Meier survival curves were plotted. Based on median mRNA expression, high and low gene expressing patient groups were created and median relapse/progression free survival probability of these two patient groups were compared. The hazard ratio (HR) was determined with 95% confidence interval and Log rank *p*-value provided the statistical significance. The number of patients at risk with the event are listed below the survival plots.

### Breast cancer patient transcriptome data analysis

To compare UBE2Q1 expression in hormone independent basal-like breast cancer patients with hormone dependent patient group, published annotated transcriptomic data from Breast Cancer Gene-Expression Miner v4.4 (http://bcgenex.centregauducheau.fr/BC-GEM) was analysed [16, 17] . A targeted expression analysis for UBE2Q1 according to PAM-50 subtypes of breast cancer was performed on DNA microarrays of pooled patient cohorts whose datasets were merged to a common scale. A Welch’s test together with Dunnett-Tukey-Kramer’s tests was performed for pairwise comparison.

### Western blotting

High grade serous ovarian cancer OVCAR8 cells and non-high grade ovarian cancer A2780 cells were cultured in RPMI media supplemented with 10% fetal bovine serum (FBS). Total cell lysates were prepared using standard RIPA buffer with proteinase inhibitor additives. The proteins were transferred to nitrocellulose membrane, blocked with 5% non-fat dry milk (NFDM) in TBST. The membrane was incubated with relevant primary and HRP conjugated anti-rabbit secondary antibodies and developed with ECL chemiluminescence procedure. Rabbit anti-human UBE2Q1 polyclonal antibody (Orb77618; Biorbyt) and Rabbit anti-human Histone H3 (ab1791; abcam) were used to detect UBE2Q1 and histone H3, respectively.

### Receiver operating characteristic (ROC) plot analysis

To examine predictive value of UBE2Q1, online validation tool ROC plotter was used. Serous ovarian cancer patient cohorts were segregated into response cohorts with the outcome of survival without disease (Responders) and disease relapse before 1 years after platin/taxane chemotherapy (Non-responders). The ROC plotter compares gene expression in responders versus non-responders and also correlates gene expression with the predictive marker value of a gene in response to treatment [18]. UBE2Q1 expression was correlated with 1-year relapse free survival of serous ovarian cancer patients in response to chemotherapeutic drugs Platin/Taxane**.** The p - value was calculated by Mann-Whitney test.

## Results

### Identification of differentially expressed genes in ovarian tumor tissues

**Fig. 1:**
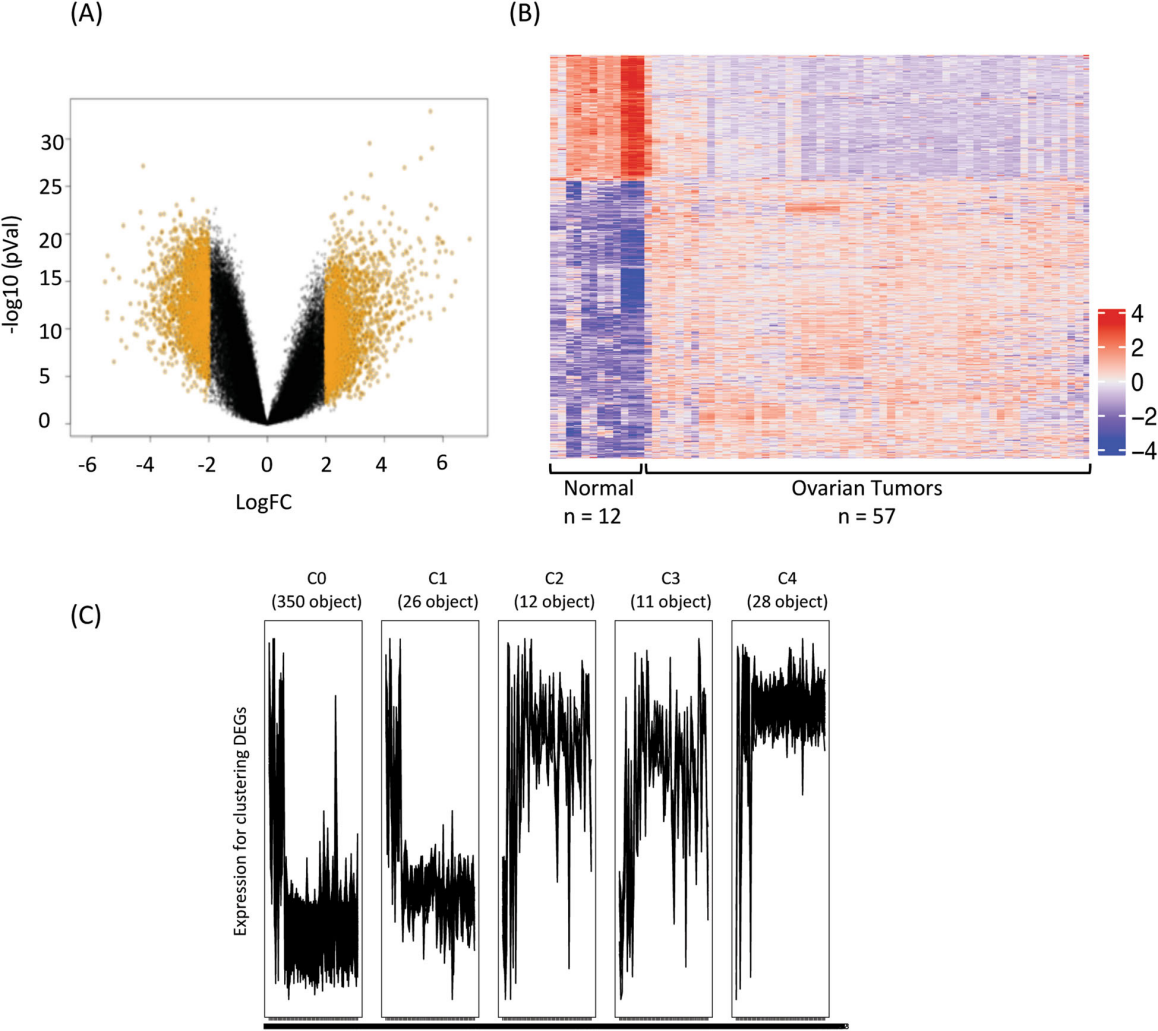
Identification of differentially expressed genes (DEGs) in ovarian cancer tissues (*n* = 57) compared to normal tissues (*n* = 12) upon analysis of microarray dataset GSE66957. **a** Volcano plot demonstrates scattering of genes that show significant differences in expression levels between primary ovarian carcinoma and normal ovarian surface epithelial tissue samples. Genes (depicted in orange) show fold change difference of > 2 and a *p*-value < 0.01. **b** Heatmap shows expression of 2607 genes that are differentially expressed in ovarian cancer in comparison to normal epithelial tissues. **c** Clustering analyses group differentially expressed genes into 5 clusters with similar patterns of expression comparing ovarian cancer tissues with normal ovarian epithelium. Gene elements of the 5 DEG clusters are provided in supplementary file 2

To identify relevant genes contributing potentially to the pathogenesis and prognosis of high grade serous ovarian cancer, we performed a genome wide expression analysis of ovarian cancer patient tumor samples (*n* = 57) and compared it with normal ovarian surface epithelial tissue samples (*n* = 12) by examining microarray data of a publicly available GEO dataset GSE66957. Keeping the cut-off stringent, genes with log2FC score difference of more than 2 in their expression were considered to be differentially expressed genes (DEGs) as illustrated in volcano plot (*p* < 0.01) (Fig. 1a). A total of 2607 differentially expressed genes (DEGs) were identified out of which 810 genes were downregulated and 1797 genes were upregulated. A list of these differentially expressed genes is provided in supplementary file 1. The expression of these differentially expressed genes was plotted as a heatmap to identify gene clusters that are differentially up/downregulated in cancer patient samples (Fig. 1b). To extract optimal co-expressed gene clusters, the DEGs were used as input to the “Clust” program. The expression data were quantile normalised and converted to z-scores as the most suitable normalisation technique for pre-processing. Clust generated 5 clusters of genes, which in total included 427 genes. The smallest cluster included 11 genes, the largest cluster included 350 genes, and the average cluster size was found to be nearly 85 genes. The 5 cluster groups were generated based on the comparable DEGs expression patterns of respective clusters in ovarian cancer tissues with a contrasting expression in normal ovarian epithelium (Fig. 1c). The clusters were divided as C0, C1, C2, C3 and C4 and multiple clustering algorithms were used to weed out data that do not fit together (Fig. 1c) and each of these clusters had 224, 19, 12, 11, 28 genes, respectively. The list of these DEGs of five clusters, altered expression of which correlated with ovarian cancer is provided in supplementary file 2.

### Weighted gene co-expression network analysis of differentially expressed genes in ovarian cancer

**Fig. 2:**
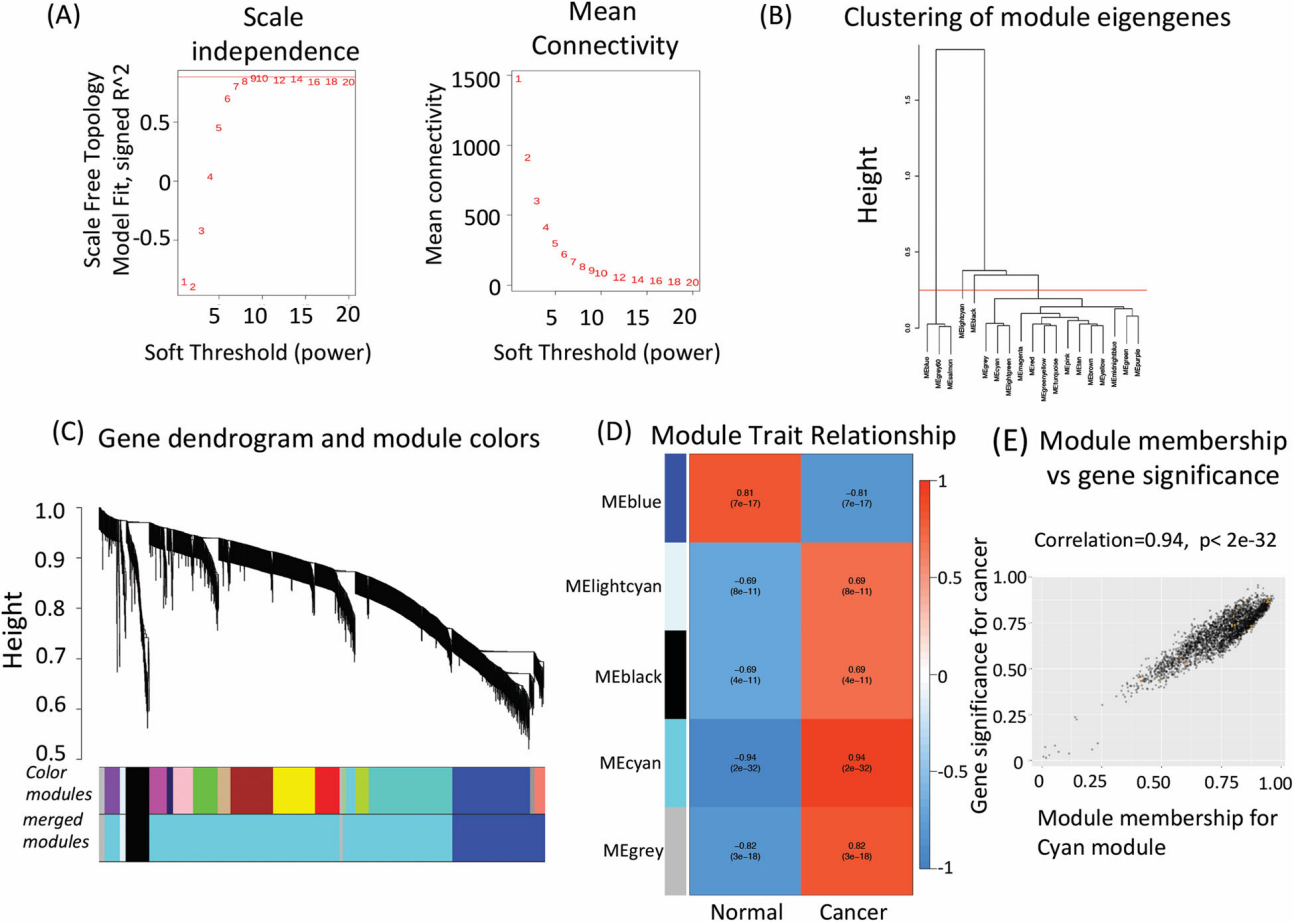
Establishing correlation patterns among differentially expressed genes (*n* = 2607) in ovarian cancer using Weighted Gene Co-expression Network (WGCNA) analysis. **a** Selection of parameter beta for the power transformation of the correlation matrix into the adjacency matrix in order to obtain scale free topology. The horizontal and vertical axes on the left plot represent the soft threshold power and the scale free fit index, respectively. The red line represents the standard line when R^2 reached a value of 0.88. The mean connectivity is depicted as a function of the soft threshold powers. **b** Similar modules with correlating module eigengenes were merged to form four major modules based on a distance threshold cut-off of 0.25. **c** Hierarchical clustering of genes with dissimilarity based on topological overlap are shown along with the modules detected and the merged modules. All genes that do not fall in any of the modules are kept in grey module. **d** Module trait relationship between cancer and normal tissues was established. Each module shows correlation coefficient and corresponding *p*-value for the correlation of genes in a specific module to the selected traits i.e. cancer or normal tissue. Cyan module showed maximum correlation and was the most relevant module with cancer traits. **e** Gene significance for ovarian cancer versus the module membership in the cyan module is depicted as a scatter plot. Intramodular analysis of the genes found in the cyan module, which contains genes that have a high correlation with ovarian cancer, with *p* < 2e-32 and correlation = 0.94

It is suggested that genes showing comparable co-expression patterns are either transcriptionally regulated in a related manner or interact functionally in similar or parallel pathways. To identify genes that are similarly regulated or fall in similar pathway in ovarian cancer patients, we also performed a weighted gene co-expression network analysis of differentially expressed genes identified in our genome wide expression analysis. Scale free topology was obtained setting the soft threshold power to 9 resulting into a R^2 value of 0.88. The mean connectivity was also analysed for the soft threshold powers (Fig. 2a). 19 modules were generated and similar modules with correlating module eigengenes were merged to generate 5 gene modules with co-expressing differentially expressed genes (Fig. 2b and c). Hierarchical clustering of genes with dissimilarity based on topological overlap matrix (TOM) facilitated construction of cluster dendrogram of gene modules (Fig. 2c). By performing a module trait relationship analysis, we identified “CYAN” module as the most relevant module harbouring 20 genes that correlated with cancer traits (Fig. 2d). Apart from cyan module, blue and black modules also showed co-expression of 107 and 20 genes, but with a lower degree of correlation in comparison to cyan module. A gene significance and module membership analyses were performed and the genes in cyan module showed a correlation cutoff of 0.94 (Fig. 2e). These genes were ZNF117/ERV3–1, ZNF117, UBE2Q1, UBE2C, TPX2, STAB2, SDHAP1, SDHAP2, TYMP, EPCAM, COL3A1, COL1A1, CLDN4, C1S, C1R, B4GALT3, TBCD, SNX25. The list of genes in black and blue modules is provided in supplementary file 3.

### Visualization network and gene ontology analysis of co-expressed genes

**Fig. 3:**
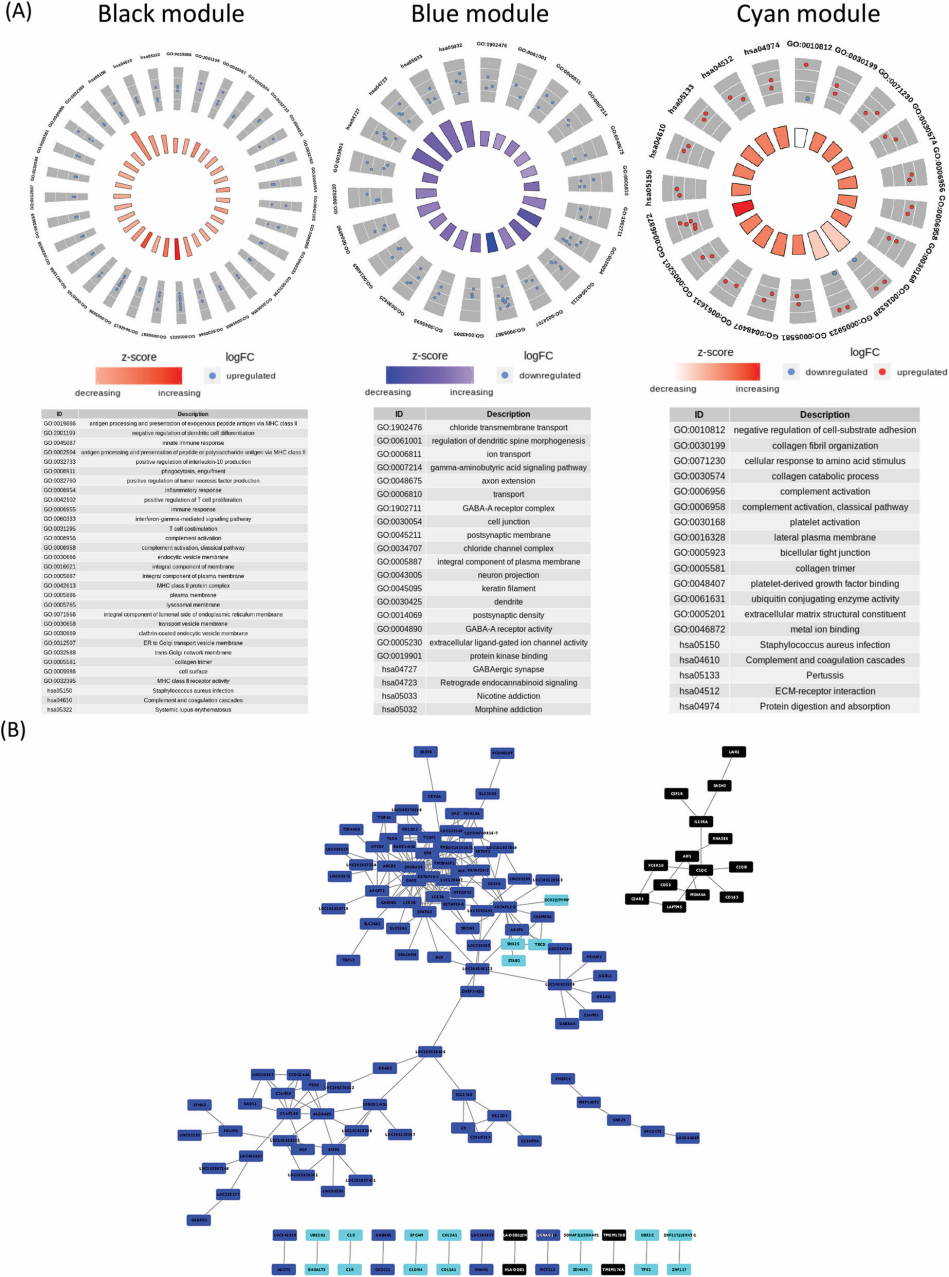
Network visualization and pathway analysis of WGCNA modules. **a** Gene enrichment terms in the biological process, cellular component, molecular function and KEGG pathways are represented for black, blue and cyan modules in circle plots and the GO terms for respective module are enlisted in the Tables. **b** Network visualization of co-expression edges in blue, black and cyan modules. Genes falling in these modules are provided in supplementary file 3

To have a better understanding of these cancer associated co-expressed genes and their functions, we performed a molecular pathway analysis for genes of cyan, blue and black modules. GO enrichment analysis was performed to determine the cellular component (CC), biological processes (BP), molecular function (MF) and KEGG pathways that are altered specifically in ovarian cancer. The pathway enrichment analyses were plotted in circle plots for respective modules and the gene enrichment (GO) terms were tabulated (Fig. 3 a and b). The list of module genes falling in each of the GO categories (BP, CC, MF, KEGG) along with the adjusted *p*-value is provided in supplementary file 3.

DEGs harbouring in black module significantly enriched in integral component of membranes, complement/coagulation cascades and *Staphylococcus aureus* infection pathways. The genes that fall in this category were HLA-DQB1, HLA-DQB2, C3AR1, C1QB, C1QC, LAIR1, IL10RA, FCER1G, CD53, MS4A6A, TMEM176B, CSF1R. Significant upregulation of these genes in ovarian cancer patient samples suggested the importance of membrane signalling and immune response including complement system in ovarian cancer pathogenesis.

DEGs harbouring in blue module were mostly downregulated and significantly enriched in KEGG pathways such as Nicotine addiction, retrograde endocannabinoid signalling, GABAergic synapse and morphine addiction. The significantly downregulated genes were GABRR3, GABRA4, GABRB1, SLC17A6, ADCY2 and NSF. All these genes associated with GABAergic signalling system, downregulation of which seems to correlate with ovarian tumorigenesis. Very little literature is available on these genes in ovarian cancer and would need further investigation in the field.

DEGs harbouring in cyan module were mostly upregulated except TBCD gene that was significantly downregulated. Cyan module showed enrichment in lateral membrane pathway including genes EPCAM, CLDN4 and TBCD in which EPCAM and CLDN4 were upregulated while TBCD was downregulated. EPCAM and Claudin 4 maintain the bicellular tight junction and their alteration is well implicated in ovarian cancer [19, 20]. Genes such as COL1A1 and COL3A1, components of collagen fibril are extracellular matrix structural constituent and play important role in cancer invasion and metastasis [21] . UBE2Q1 and UBE2C share their molecular function as ubiquitin conjugating enzyme activity with role in protein turnover, which is altered in many cancers but not yet implicated in ovarian cancer.

The co-expression edges correlating the genes of blue, black and cyan modules are depicted in visualization network (Fig. 3b). In module trait relationship (Fig. 2d), we found that the genes in cyan module have maximum co-expression correlation and also strongest association with the cancer trait. Hence, in this visualization network, we focussed on the co-expression patterns of genes of cyan module and their relationship with ovarian cancer. Seven gene pairs of cyan modules showed significant co-expression relationship and might have functional interaction and implication in ovarian cancer. These identified pairs were UBE2Q1 – B4GALT3, C1S - C1R, EPCAM-CLDN4, COL3A1 – COL1A1, SDHAP2/SDHAP1 – SDHAP1, UBE2C – TPX2, ZNF117 /ERV3–1 – ZNF117. The other cyan module genes namely SNX25, STAB2, TBCD, SCO2/TYMP showed inter-relationship and also correlated with the downregulated genes of blue module.

### UBE2Q1 is a potential prognostic and predictive marker for relapse free survival of serous ovarian cancer patients

To identify overlapping candidates from DEG and WGCNA analysis, we searched for genes that were common in cluster C4 of DEG “clust” analysis and cyan module obtained by WGCNA. Cluster C4 contains genes with stringent similar expression patterns while cyan module genes showed maximum expression correlation. Gene overlapping of cluster4 and cyan module identified only two genes “UBE2Q1” and “B4GALT3” that were co-expressing as visualised by the network analysis. To understand the importance of these genes in ovarian cancer pathogenesis, we analysed the impact of overexpression of these two genes on relapse free survival of ovarian cancer patients using Kaplan Meier survival plots and the level of genetic alteration observed for these genes in TCGA database [22] . Nearly 20% alteration frequency (including mainly copy number amplification and mRNA overexpression) was observed for UBE2Q1 – B4GALT3 gene pair and they were chosen for further analysis.

**Fig. 4:**
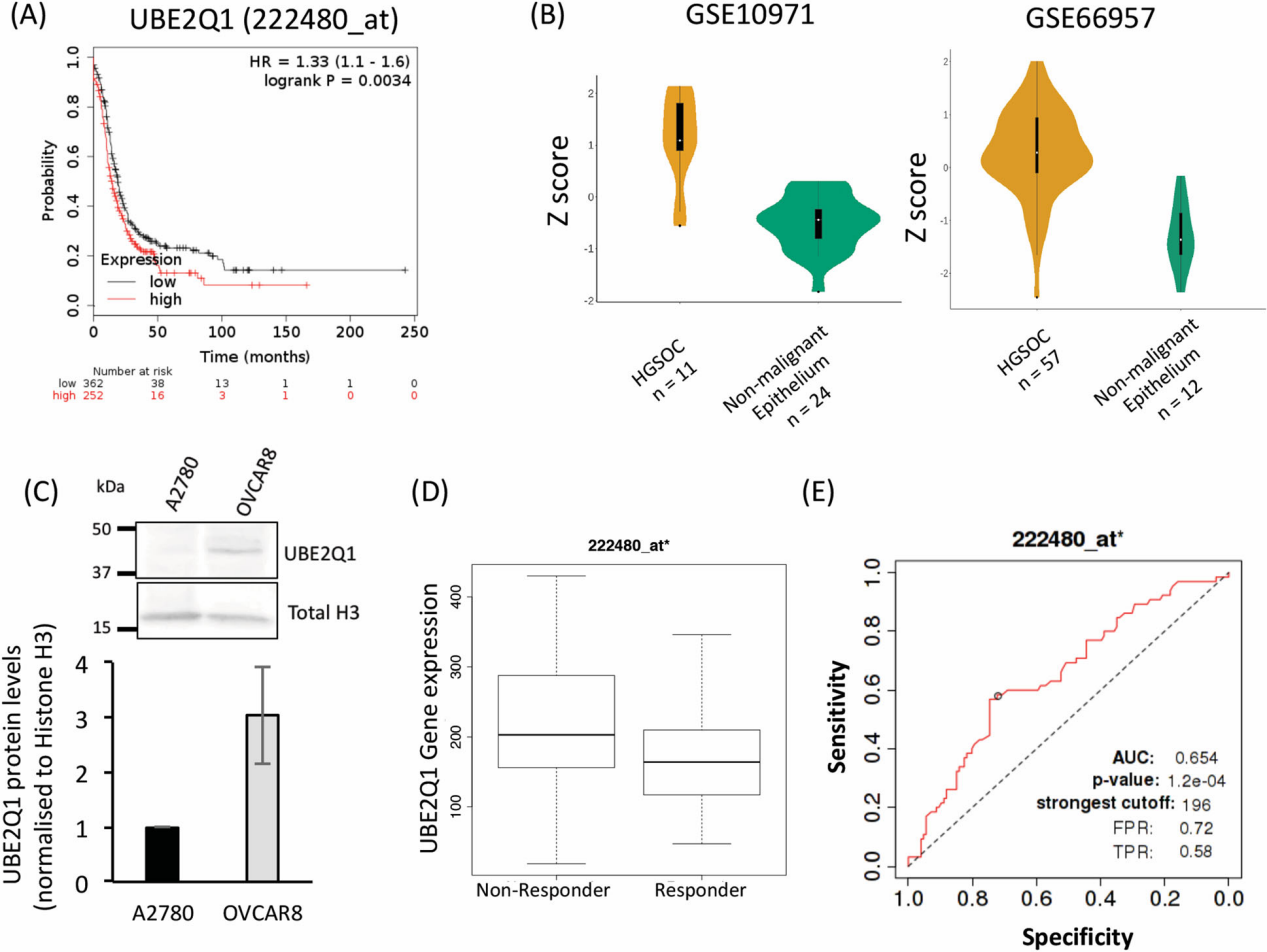
Identification of UBE2Q1 as a poor prognostic and predictive marker for high grade serous ovarian carcinoma. **a** Kaplan Meier survival plots correlate high expression of UBE2Q1 (Probe id = 222480_at) with reduced relapse free survival probability of ovarian cancer patients with hazard ratio of 1.33 (**b**) Increased expression of UBE2Q1 was observed in tumor samples of high grade serous ovarian cancer patients (*n* = 11) compared to non-malignant fallopian tube epithelium tissues (*n* = 24) using validation dataset GSE10971. The UBE2Q1 levels in experimental dataset GSE66957 is also plotted for comparison. **c** Western blot analysis depicts increase in levels of UBE2Q1 in OVCAR8 high grade serous ovarian cancer cells compared to A2780 cells. Total Histone H3 was used as loading control. The uncropped Western blot images are provided in supplementary Fig. 3. The densitometric analysis of Western blots was performed using ImageJ. **d** The serous ovarian cancer patient cohorts (grade III) were treated with combination of platin/taxane and the response outcomes were determined by relapse free survival over 12 months. UBE2Q1 expression was significantly higher in non-responders (*n* = 65) to platin/taxane drug treatment when compared to responders (*n* = 126); *p-*value was 0.00051 as calculated by Mann-Whitney test. **e** ROC plot illustrates the predictive biomarker value of UBE2Q1 in response to platin/taxane therapy with AUC value equals to 0.654 (*p* value = 1.2e-04)

To investigate the potential of UBE2Q1 and B4GALT3 as prognostic markers, we performed survival analysis on these genes to identify their effects on relapse free survival of ovarian cancer patients. We observed that patients with high-UBE2Q1 expression showed poor relapse-free survival compared to low-UBE2Q1 expressing patients (Fig. 4a). However, only a marginal effect was observed on patient’s survival upon B4GALT3 overexpression (Supplementary Fig. 1; A). As major prognostic effect on patients was observed for UBE2Q1, we focussed on this gene. The UBE2Q1 protein architecture consists of 5 alpha helices, 6 beta chains, a C-terminus UBC catalytic domain (Supplementary Fig. 2A-C) [23, 24]. We further analysed the expression of UBE2Q1 gene in experimental GEO dataset GSE66957 which indeed showed significant upregulation of UBE2Q1 in high grade serous ovarian cancer patients in comparison to normal ovarian surface epithelium (Fig. 4b). Further we validated this result in another dataset GSE10971 termed as ‘validation dataset’ and also found increased expression of UBE2Q1 in tumor samples of high grade serous ovarian cancer patients (*n* = 11) compared to non-malignant fallopian tube epithelium tissues (*n* = 24) (Fig. 4b). To corroborate this finding at cellular level, we analysed UBE2Q1 protein expression in high grade serous ovarian cancer cells (OVCAR8) in comparison to non-high grade ovarian cancer cells (A2780). Significant increased expression of UBE2Q1 (*p* < 0.05) was observed in high grade serous ovarian cells compared to A2780 cells (Fig. 4c). Further, to assess prognostic and predictive value of UBE2Q1 in ovarian cancer patients post chemotherapy with paclitaxel and platinum drugs, we utilized a publicly available online analysis tool ROC Plotter (www.rocplot.org/ovar), which analysed chemotherapy response using clinical data of cancer patients obtained from GEO and TCGA repositories and facilitate predictive marker validation in ovarian cancer after various therapies [18] and correlated it with gene expression. Ovarian cancer patients’ data that were histologically grade 3 serous type were used for this analysis to correlate UBE2Q1 with aggressive subtype of ovarian cancer. This patient cohort (*n* = 191) was treated with combination of taxane and platin based drugs and subdivided into two groups, responders (*n* = 126) and non-responders (*n* = 65). The response outcome was based on the relapse free survival (RFS) within a recovery period of 12 months for responders whereas, patients whose disease relapsed after the combination drug treatment within a period of 12 months were designated as non-responders. The expression of UBE2Q1 was evaluated in both cohorts and plotted as boxplot (Fig. 4d). We found that non-responders expressed higher levels of UBE2Q1 compared to responders (Mann-Whitney test; *p*-value = 0.00051) upon treatment with platin/taxane combination. To establish clinical utility of UBE2Q1, receiver operating characteristic (ROC) plot were generated and area under curve (AUC) was calculated using online open resource tool (ROCplotter.com) (Fig. 4e). An AUC value of 0.654 (*p* value = 1.2e-04) suggests a convincing potential of UBE2Q1 as a predictive biomarker of clinical utility to predict relapse free survival of serous ovarian cancer patients of later stages/grades in response to platin/taxane therapy.

### UBE2Q1 is a potential prognostic factor for hormone independent breast cancer patients

**Fig. 5:**
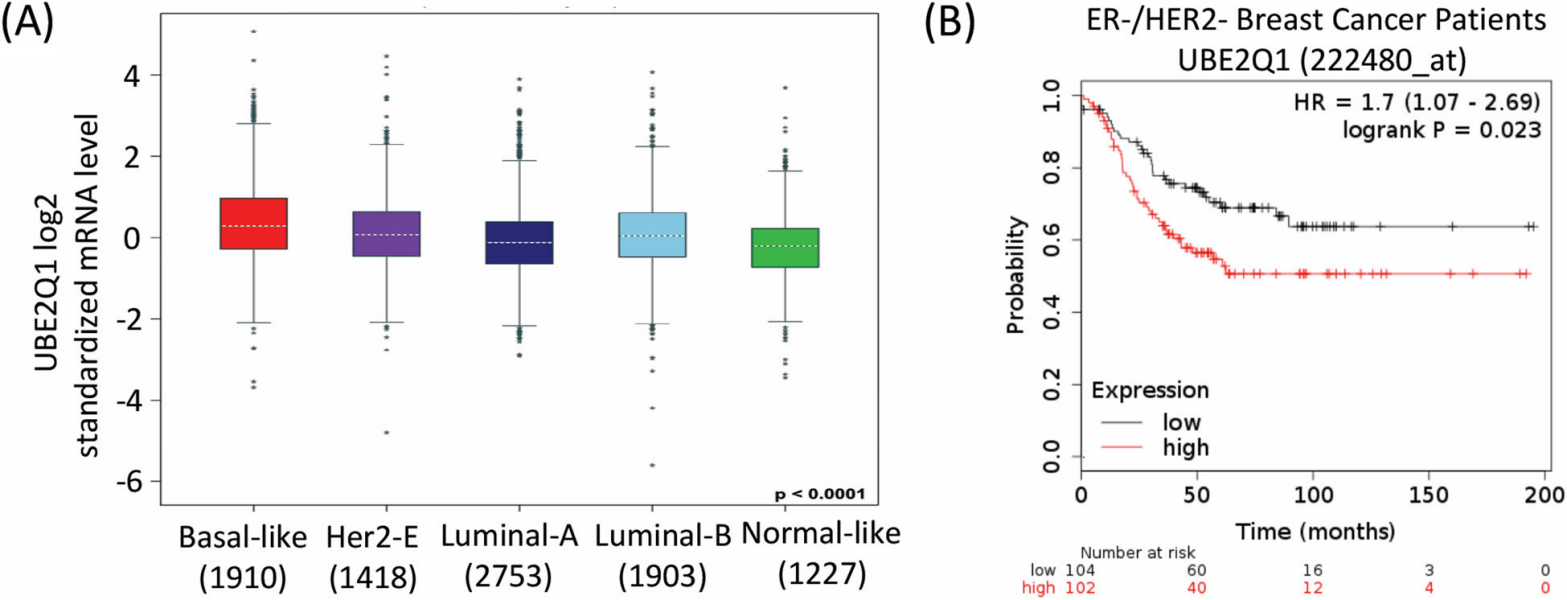
UBE2Q1 functions as a poor prognostic marker for hormone independent ER−/HER2- breast cancers. **a** Increased mRNA expression of UBE2Q1 in tumor samples of hormone independent basal-like breast cancer patients in comparison to HER2- enriched (HER2-E), Luminal-A, Luminal-B and normal-like hormone dependent breast cancer subtypes (p value < 0.0001). **b** Kaplan Meier survival plots correlate high expression of UBE2Q1 (Probe id = 222480_at) with reduced probability of relapse free survival of hormone independent ER−/HER2- breast cancer patients

Analogous to ovarian cancer, breast cancer shares similar genetic and epigenetic alterations and involves alterations in female hormones. Hence, we investigated the expression of UBE2Q1 in different subtypes of breast cancer categorised based on 3-Gene classifiers subtypes i.e. Basal like, Her2-enriched, Luminal A, Luminal B and normal-like. Strikingly, a significant overexpression of UBE2Q1 was observed in tumor samples of hormone independent basal-like breast cancer patients, the most aggressive subtype, in comparison to hormone dependent non-basal-like breast cancers (p value < 0.0001) (Fig. 5a). Notably, high expression of UBE2Q1 correlated with poor relapse free survival of hormone independent ER−/HER2- breast cancer patients (Fig. 5b). This is in line with our observation that even in Ovarian cancer, it is the aggressive serous subtype which shows poor prognosis in correlation with high UBE2Q1 expression.

In the WGCNA network (Fig. 3b), UBE2Q1 showed a co-expression correlation with B4GALT3. We validated this correlation in TCGA dataset using ovarian U133 microarray data and in three breast cancer datasets from BCGeneMiner, an online open source tool (http://bcgenex.centregauducheau.fr/BC-GEM/GEM-Accueil.php?js=1). We indeed observed co-expression correlation of UBE2Q1 with B4GALT3 in a moderate level (Supplementary Fig. S1; C & D). We also evaluated for the consequence of high expression of B4GALT3 on breast cancer patient survival and alike ovarian cancer, no significant adverse effect was observed for breast cancer patients expressing high B4GALT3 (Supplementary Fig. S1; B).

Thus, our study identifies a potential UBE2Q1 – B4GALT3 functional axis in breast and ovarian cancer, where only the E2 conjugating enzyme showed a poor prognostic impact on the disease. We, for the first time identify UBE2Q1 as a potential prognostic marker for aggressive ovarian and breast cancers and predictive marker for platin/taxane chemotherapy in high grade serous ovarian carcinoma.

## Discussion

In this study, we have identified UBE2Q1 as a potential prognostic marker for ovarian cancer. By performing genome wide expression analysis of tumor samples of high grade serous ovarian cancer patients and generating co-expression patterns of differentially expressed genes (DEGs), we found UBE2Q1 - B4GALT3 axis as an important player in female reproductive cancers. WGCNA analysis identified cyan module as the most relevant module to the cancer traits comprising 20 genes using a stringent correlation cut-off of > 0.9. Out of these genes, increased expression of UBE2Q1 showed negative impact on the 5-year relapse free survival of ovarian cancer patients and also 1-year relapse free survival after platin/taxane chemotherapy in grade III serous ovarian cancer patients. Considering etiological similarities in ovarian and breast cancer, we compared expression of UBE2Q1 in different breast cancer subtypes and observed highest expression in most aggressive basal-like breast cancer patients, which are essentially negative for HER2 and estrogen receptors. This enhanced expression also correlated with a poor prognosis in ER−/HER2- breast cancer patients. Further, both in ovarian and ER−/HER2- breast cancer patients, a co-expression pattern for UBE2Q1 alongwith B4GALT3 was observed suggesting a functional interaction and potential pathological contribution of these two genes in the cancer subtypes. Functionally, both UBE2Q1 and B4GALT3 are post translational modulators of proteins. As an E2 ubiquitin conjugating enzyme, UBE2Q1 mediates protein ubiquitination targeting the substrate for proteasomal degradation while B4GALT3, one of the B4GALT family members, performs protein glycosylation mostly functioning as protein stabilizer. Co-alteration of these genes suggest that mis-regulation in protein turnover and stability plays a key role in ovarian tumorigenesis.

Although increased expression of UBE2Q1 has been observed in various cancers such as breast cancer, colorectal cancer and hepatocellular carcinoma [25–27]**,** no previous report has explored this gene in ovarian cancer to best of our knowledge**.** UBE2Q1 has been identified as a poor prognosticator for hepatocellular carcinoma and early stage laryngeal cancers [28, 29]. Another study analysed promoter methylation of UBE2Q1 in serum of hepatocellular carcinoma (HCC) patients and demonstrated low levels of UBE2Q1 DNA methylation as a potential biomarker for hepatitis B-virus (HBV) positive HCC [30]. Notably, DNA hypomethylation is associated with transcriptional activation of the gene. Eventhough transcript and/or protein levels of UBE2Q1 were not tested in this study; high levels of UBE2Q1 have been associated with poor prognosis in HCC patients [27]. Overexpressing UBE2Q1 in colorectal cancer cell line showed an oncogenic function of the gene inducing proliferation and migration of tumor cells [31]. UBE2Q1 may regulate various protein substrates by ubiquitination and functionally may lead to instability of tumor suppressor genes in cancers. One such report showed instability of p53 upon UBE2Q1 overexpression, but the exact mechanism is still unexplored [32]. The involvement of process of protein ubiquitination is well documented in regulation of steroid hormone homeostasis. For example, the ubiquitin protein ligase E3A (UBE3A) acted as an coactivator for steroid hormone [33]. Moreover, an altered expression whether deletion or overexpression of UBE3A dysregulated levels of estrogen and progesterone receptors [34]. It is noteworthy that UBE2Q1 knockout female mice demonstrate irregular oestrus cycle and abnormal mating behavior which might indicate aberrant steroid hormone signaling [13]. Thus, we propose a potential role of UBE2Q1 in hormonal homeostasis and a deeper understanding of this function in female reproductive system might give better clues on how UBE2Q1 contributes to female reproductive cancers.

We also identified β-1,4-galactosyltransferase III (B4GALT3) as a co-expressing gene of UBE2Q1, which is suggested as integrating key functional player. We have observed differential overexpression of B4GALT3 and also a co-expressional pattern with UBE2Q1, however it does not correlate with ovarian and breast cancer prognosis. Notably, this gene behaves differently in other malignancies. In cervical epithelial cancer, overexpression of B4GALT3 is observed which increases migration, invasion and EMT of tumor cells [35]. Another study in neuroblastoma, the authors showed similar phenomenon and suggested that this function of B4GALT3 is driven by glycosylation of integrin beta 1 resulting in extended retention of matured beta 1 integrin and increased phosphorylation of focal adhesion kinases (p-FAK) consequently leading to enhanced tumor cell migration and invasion [36]. Even in glioblastoma, overexpression of B4GALT3 is associated with poor prognosis of patients and suggest this protein as oncogenic in nature [37]. Thus, we infer that although B4GALT3 has no impact on ovarian cancer prognosis, its potential oncogenic function could not be excluded and would need further investigation. Moreover, the first member of B4GALT family, B4GALT1 has been partially studied in ovarian cancer invasion and metastasis [38, 39] . Thus, it would be interesting to investigate exact function of B4GALT3 in ovarian cancer in future.

Our in-silico gene co-expression network analysis has identified certain genes that are yet not implicated in ovarian cancer disease. One of the genes identified in Cyan module was ERV3 – ZNF117. This protein coding readthrough transcript of ERV3 (endogenous retrovirus group 3) and zinc finger protein 117 (ZNF117) codes for the same protein as ZNF117 protein [40]. Interestingly, we observed that enhanced expression of this protein is associated with poor relapse free prognosis of ovarian cancer patients (data not shown). However, role of this protein is not studied even though 30% antibodies to ERV3 is observed in ovarian cancer patients [40]. Thus, our study identifies a new gene with potential implication in ovarian cancer and require a detailed study to unravel its function. Notably, in blue module, we observed downregulation of number of genes involved in GABAergic signalling. Recent studies have identified neurotransmitters as emerging therapeutic targets in cancer [41]. GABAergic signalling plays important role in immune system and inflammatory response and may function as anti-tumor molecules, thus a study of these genes might identify novel neurotransmitters as potential therapeutic targets in ovarian cancer.

## Conclusions

To conclude, we find UBE2Q1 as a potential prognostic marker for ovarian cancer patients’ post-chemotherapy with platin/taxane drugs. We also propose, a UBE2Q1 – B4GALT3 axis, co-relative overexpression of which is envisaged to have an oncogenic potential in ovarian cancer development. Further, experimental analyses of these genes would be performed in future to confirm their oncogenic roles in ovarian cancer.

## Supplementary Information

**Additional file 1: Supplementary file 1:** List of 2607 differentially expressed genes identified from microarray data analysis.

**Additional file 2: Supplementary file 2:** Gene elements in each of the five differentially expressed gene clusters having similar expression patterns is depicted here.

**Additional file 3: Supplementary file 3:** Genes falling in the black, blue and cyan modules are represented here.

**Additional file 4: Supplementary Fig. 1:** (A & B) Kaplan Meier survival plots showing moderate to no correlation of high expression of B4GALT3 (Probe id = 210243_s_at) with relapse free survival probability of ovarian (A) and breast (B) cancer patients. (C & D) Co-expression correlation of UBE2Q1 with B4GALT3 in ovarian cancer (TCGA dataset) and basal-like breast cancer (BCGeneMiner-3 datasets) patient samples.

**Additional file 5: Supplementary Fig. 2:** (A) Structure of UBE2Q1 depicting UBC catalytic domain at the C terminus and a site of protein modification (red dot) (HPRD ID: 15601) (B & C) 3D structure of UBE2Q1 showing 5 alpha helices and 6 beta strands (PDB ID 2QGX).

**Additional file 6: Supplementary Fig. 3:** Uncropped images of the Western blots provided in Fig. 4c.

## Data Availability

The microarray datasets used in the current study are available in the ArrayExpress database at EMBL-EBI (www.ebi.ac.uk/arrayexpress) under accession number E-GEOD-66957 and E-GEOD-10971. All data generated or analysed during this study are included in this published article and its supplementary information files 1, 2 and 3 and any other information are available from the corresponding authors on request.

## Abbreviations

HGSOC: High Grade Serous Ovarian Cancer
UBE2Q1: Ubiquitin-conjugating enzyme E2 Q1
B4GALT3: β-1,4-galactosyltransferase III
DEG: Differentially expressed genes
WGCNA: Weighted gene coexpression network analysis
GEO: Gene Expression Omnibus
TCGA: The Cancer Genome Atlas
KEGG: Kyoto Encyclopedia of Genes and Genomes
TOM: Topological overlap matrix
CC: Cellular component
BP: Biological processes
MF: Molecular function
ER−/HER2-: Estrogen and HER2 receptor negative
RFS: Relapse free survival
