# Supplementary material for: Integrated bioinformatic analysis identifies UBE2Q1 as a potential prognostic marker for high grade serous ovarian cancer
Source: BMC Cancer. 2021 Mar 4;21:220. doi: 10.1186/s12885-021-07928-z (PMC7934452; doi:10.1186/s12885-021-07928-z)
Supplement: Supplementary file 4 — Additional file 4: Supplementary Fig. 1: (A & B) Kaplan Meier survival plots showing moderate to no correlation of high expression of B4GALT3 (Probe id = 210243_s_at) with relapse free survival probability of ovarian (A) and breast (B) cancer patients. (C & D) Co-expression correlation of UBE2Q1 with B4GALT3 in ovarian cancer (TCGA dataset) and basal-like breast cancer (BCGeneMiner-3 datasets) patient samples. [file 12885_2021_7928_MOESM4_ESM.pdf]

Supplementary figure 1: (A & B) Kaplan Meier survival plots showing moderate to no correlation of high expression of B4GALT3 (Probe id = 210243_s_at) with relapse free survival probability of ovarian (A) and breast (B) cancer patients. (C & D) Co-expression correlation of UBE2Q1 with B4GALT3 in ovarian cancer (TCGA dataset) and basal-like breast cancer (BCGeneMiner-3 datasets) patient samples.

(A)

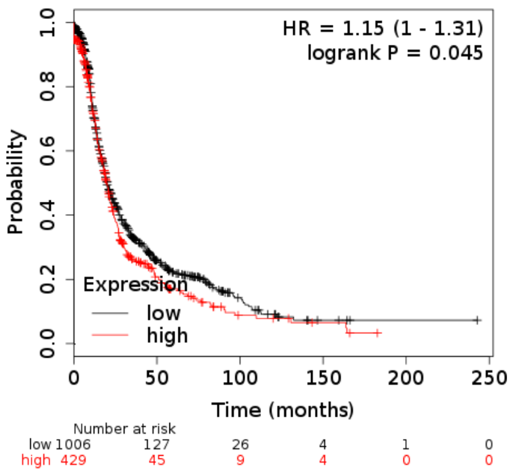

(B)

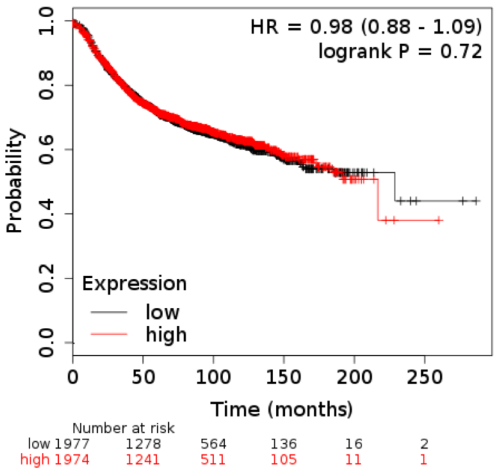

(C)

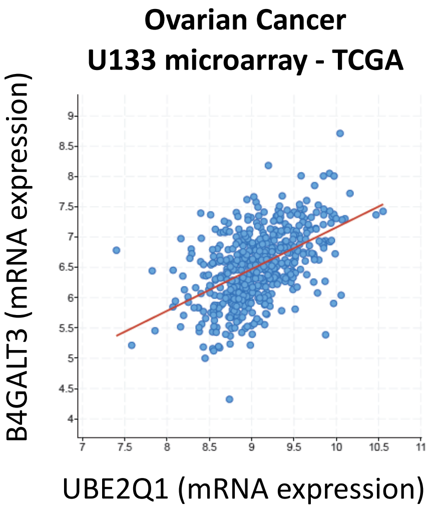

Pearson correlation: r = 0.50
Sample no. = 530
p value = 4.75e -35

(D)

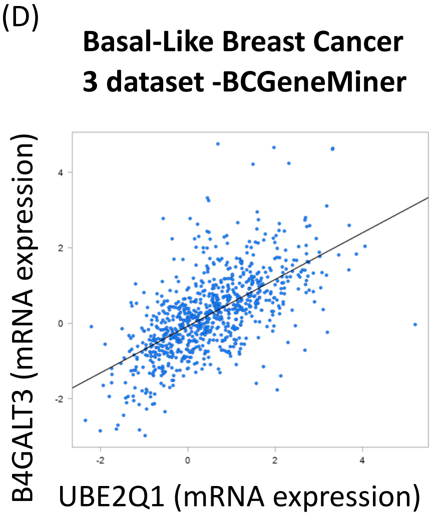

Pearson correlation: r = 0.62
Sample no. = 832
p value < 0.0001
