# Supplementary material for: Integrated bioinformatic analysis identifies UBE2Q1 as a potential prognostic marker for high grade serous ovarian cancer
Source: BMC Cancer. 2021 Mar 4;21:220. doi: 10.1186/s12885-021-07928-z (PMC7934452; doi:10.1186/s12885-021-07928-z)

Supplementary figure 2: (A) Structure of UBE2Q1 depicting UBC catalytic domain at the C terminus and a site of protein modification (red dot) (HPRD ID: 15601) (B & C) 3D structure of UBE2Q1 showing 5 alpha helices and 6 beta strands (PDB ID 2QGX).

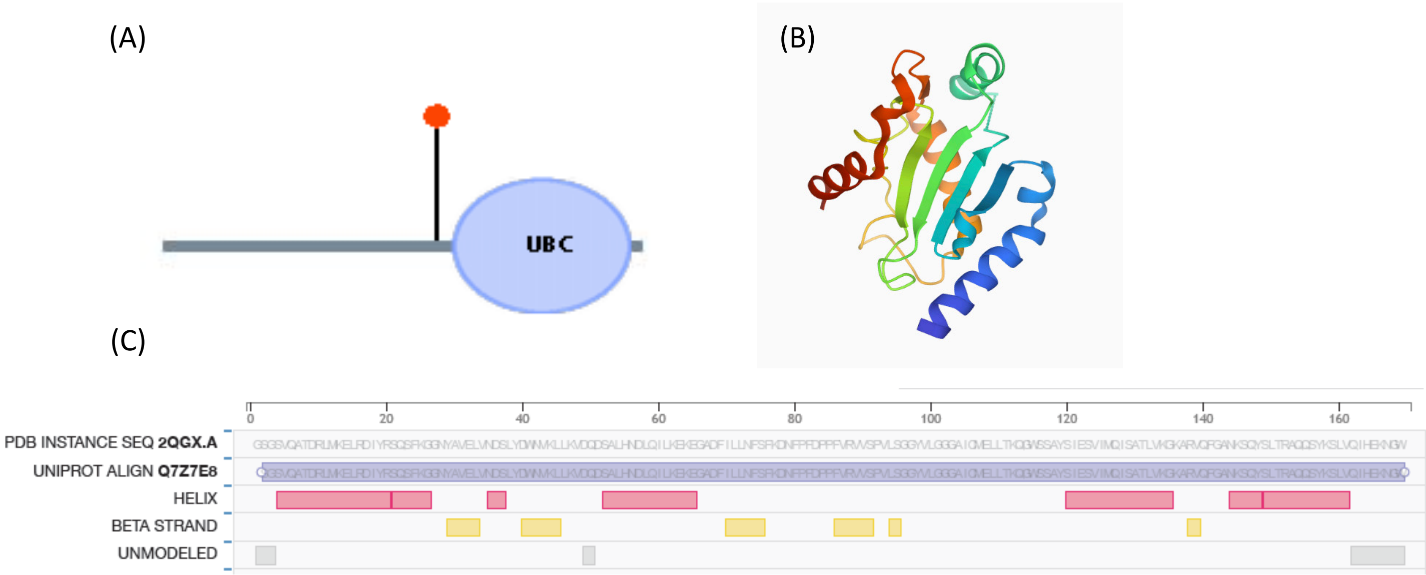

Supplement: Supplementary file 5 — Additional file 5: Supplementary Fig. 2: (A) Structure of UBE2Q1 depicting UBC catalytic domain at the C terminus and a site of protein modification (red dot) (HPRD ID: 15601) (B & C) 3D structure of UBE2Q1 showing 5 alpha helices and 6 beta strands (PDB ID 2QGX). [file 12885_2021_7928_MOESM5_ESM.pdf]
