# Supplementary material for: Integrated bioinformatic analysis identifies UBE2Q1 as a potential prognostic marker for high grade serous ovarian cancer
Source: BMC Cancer. 2021 Mar 4;21:220. doi: 10.1186/s12885-021-07928-z (PMC7934452; doi:10.1186/s12885-021-07928-z)

Supplementary figure 3: Uncropped images of western blots of figure 4C. Exp 1 and 2 demonstrates two biological replicates. The rectangles show the cropped areas of the blots.

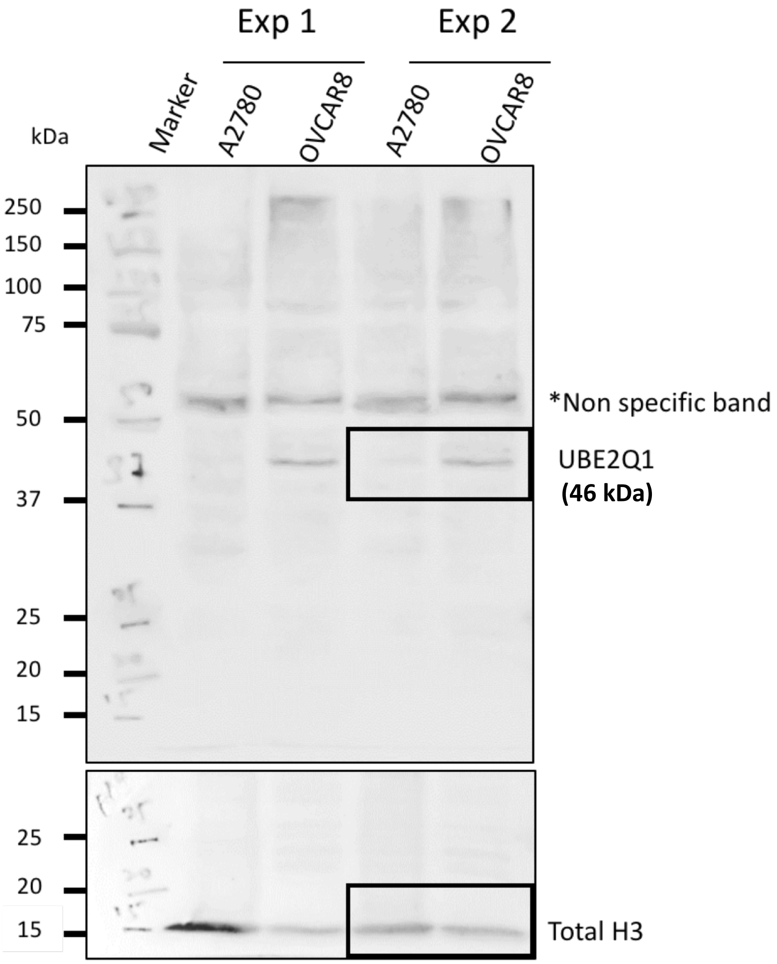

Supplement: Supplementary file 6 — Additional file 6: Supplementary Fig. 3: Uncropped images of the Western blots provided in Fig. 4c. [file 12885_2021_7928_MOESM6_ESM.pdf]
